# The potential roles of type I interferon activated neutrophils and neutrophil extracellular traps (NETs) in the pathogenesis of primary Sjögren’s syndrome

**DOI:** 10.1186/s13075-022-02860-4

**Published:** 2022-07-19

**Authors:** Yu Peng, Xunyao Wu, Shulan Zhang, Chuiwen Deng, Lidan Zhao, Mu Wang, Qingjun Wu, Huaxia Yang, Jiaxin Zhou, Linyi Peng, Xuan Luo, Yingying Chen, Anqi Wang, Qiufeng Xiao, Wen Zhang, Yan Zhao, Xiaofeng Zeng, Yunyun Fei

**Affiliations:** 1 Department of Rheumatology, Peking Union Medical College Hospital, Chinese Academy of Medical Science & Peking Union Medical College, National Clinical Research Center for Dermatologic and Immunologic Diseases, State Key Laboratory of Complex Severe and Rare Diseases, #1 Shuai-Fu-Yuan, Dongcheng District, Beijing, 100730 China; 2 Clinical Biobank, Department of Medical Research Center, Peking Union Medical College Hospital, Chinese Academy of Medical Sciences and Peking Union Medical College, Beijing, China; 3 Department of Stomatology, Peking Union Medical College Hospital, Chinese Academy of Medical Sciences & Peking Union Medical College, No.1 Shuaifuyuan, Wangfujing, Dongcheng District, Beijing, 100730 China

**Keywords:** Primary Sjögren’s syndrome, Neutrophils, Neutrophil extracellular traps, Type I interferon, Mitochondrial damage

## Abstract

**Objective:**

Neutrophils and aberrant NETosis have been implicated in the pathogenesis of diverse autoimmune diseases; however, their roles in primary Sjögren’s syndrome (pSS) remain unclear. We aimed to reveal the potential roles of neutrophils and neutrophil extracellular traps (NETs) in pSS.

**Methods:**

pSS patients were enrolled and NETosis markers were measured in plasma and labial glands using ELISA and immunofluorescence. The gene signatures of neutrophils were assessed by RNA-Seq and RT-PCR. Reactive oxygen species (ROS), mitochondrial ROS (MitoSOX) production, and JC-1 were measured by flow cytometry.

**Results:**

NETosis markers including cell-free DNA (cf-DNA) and myeloperoxidase (MPO) in plasma and labial glands from pSS patients were significantly higher than healthy controls (HCs) and were associated with disease activity. RNA sequencing and RT-qPCR revealed activated type I IFN signaling pathway and higher expression of genes related to type I interferon in pSS neutrophils. Further stimulating with IFN-α 2a in vitro significantly induced ROS production and JC-1 monomer percentage in pSS neutrophils.

**Conclusions:**

Our data suggest the involvement of neutrophils and enhanced NETosis in pSS patients. Further mechanism study in vitro revealed that type I IFN activation in pSS neutrophils led to mitochondrial damage and related ROS production which finally result in the generation of NETs.

**Supplementary Information:**

The online version contains supplementary material available at 10.1186/s13075-022-02860-4.

## Background

While considered as the first line to defend against invading pathogens, neutrophils have recently been found involved in the pathogenesis of autoimmune diseases including systemic lupus erythematosus (SLE) [1], rheumatoid arthritis (RA) [2], and ANCA-associated vasculitis (AAV) [3]. One essential mechanism is known as NETs that contribute to normal tissue damage during autoimmune conditions [4].

NETs are released after a programmed cell death processing called NETosis and are composed of neutrophil chromatin decorated with histones, protease, granular, and cytosolic proteins including myeloperoxidase (MPO) and neutrophil elastase (NE). Granular protein like MPO is an indispensable part of NETs and an eight-protein complex, which contains three highly homologous granules and resides in azurophilic granules and is small enough to pass through the nuclear pore and clip histones, which can facilitate chromatin relaxation [5]. The formation of NETs is an amplification of neutrophil function, the extracellular structure forms a physical barrier to prevent the further spread of infection and the proteases decorated on it can kill pathogens [6].

However, NET components released through NET formation could break immune tolerance and promote autoantibodies production [2, 7]*.* The autoantibody production could inverse activate neutrophils and lead to the reactive oxygen species (ROS) production and further NET formation [8]. In SLE, NET component anti-microbial peptides (LL-37 and HMGB1) could activate plasmacytoid dendritic cells (pDC) through Toll-like receptor (TLR)-9 signaling pathways and promote type I IFN secretion [9]. Neutrophils are the main cell type infiltrating in synovial fluid of RA patients [10], and NETosis take place at the site of inflammation. Peptidyl arginine deiminase enzymes 4 (PAD4) play a central role in the NET formation of RA patients and could be a source of citrullinated proteins [11]. NET components could be sensed by fibroblast-like synoviocytes (FLS) in glycation end-product (RAGE)-TLR9-dependent manner and further presented to CD4^+^ T cells, leading to the subsequent auto-inflammatory response in synovial tissue of RA patients [12].

Primary Sjögren’s syndrome (pSS) is a multifactorial systemic autoimmune disease and characterized by exocrine glands structure damage and their impaired function [13]. Abnormally activated immune cells in peripheral blood or infiltration into salivary gland tissues contributed to disease pathogenesis in pSS patients. So far, there is still no literature reporting about whether neutrophils and associated NETs and ROS production are involved in the immune pathogenesis of pSS patients. Therefore, in this study, we analyzed the neutrophils and detailed mechanisms in pSS patients. Our present findings firstly address a potential contributing role for neutrophil in the pSS.

## Materials and methods

### Study design

All the pSS patients were enrolled in Peking Union Medical College Hospital (PUMCH) and met the American-European consensus (AECG) criteria [14]. All the enrolled pSS patients were treatment-naïve and did not receive glucocorticoid (GCs) or immunosuppressant treatment before blood collection. Patients accompanied with other autoimmune diseases and malignant diseases were not enrolled in this study. ESSDAI was used to estimate patients’ disease activity in this study [15]. Labial glands from pSS patients and controls (patients with xerostomia and did not meet the diagnosis of pSS) were obtained via the lip biopsy completed by the Department of Stomatology, Peking Union Medical College Hospital, and the labial gland tissue used in our study was the remaining tissue from the pathological examination. Patients/healthy controls have all signed informed consent. This study was approved by the Ethics committee of Peking Union Medical College hospital (*Approval number: JS-3035*) and was performed according to the declaration of Helsinki. All methods are conducted in accordance with the relevant guidelines. The clinical features of patients and healthy controls are summarized in Table 1.

**Table 1:** Summary for the clinical features of the pSS patients and healthy controls enrolled in this study

Clinical features	pSS patients (***n***=50)	Healthy controls (***n***=44)
Sex (female)	49	43
Age (Mean±S.D.)	48.19±15.13	43.85±11.35
Disease duration (median, IQR)	24 (6~48)	N/A
Fever (*n*%)	2 (4.00%)	0
Joint pain (*n*%)	17 (34.00%)	0
Extra-glandular organ involvement	19 (38.00%)	0
ESSDAI (Mean±S.D.)	4.2±3.1	N/A
Serological examinations (Median, IQR)		
IgG (g/L)	18.74 (13.85~26)	N/A
IgA (g/L)	2.85 (1.84~3.41)	N/A
IgM (g/L)	1.16 (0.74~1.38)	N/A
C3 (g/L)	1.05 (0.91~1.15)	N/A
C4 (g/L)	0.17 (0.15~0.21)	N/A
RF (IU/ml)	106.5 (18.75~236.75)	N/A
ESR (mm/h)	17 (11~41.75)	N/A
CRP (mg/L)	0.79 (0.31~1.75)	N/A
Anti-SSA antibody (*n*%)	49 (98.00%)	0
Anti-SSB antibody (*n*%)	23 (46.00%)	0
Anti-Ro52 antibody (*n*%)	39 (78.00%)	0

Age for pSS patients represented for age at onset, and for healthy controls represented for the age at which the blood samples were collected. *ESSDAI* represented for EULAR primary Sjögren’s syndrome disease activity index and was used to evaluate the disease activity of pSS patients

*S.D.* standard deviation, *IQR* interquartile range, *N/A* not applicable, *ESR* erythrocyte sedimentation rate, *CRP* C-reaction protein, *IgG, IgA, IgM* immunoglobulin G, immunoglobulin A, immunoglobulin M, *C3, C4* complement 3, complement 4, *RF* rheumatic factor, *ANAs* antinuclear antibodies

### Identification of low-density granulocytes (LDGs) and purification of neutrophils

Peripheral blood collected in the ethylene diamine tetraacetic acid (EDTA) tubes was 1:1 diluted with phosphate buffer saline (PBS) buffer, laid on the Ficoll density gradient (DAKEWE, China), and then centrifuged at 1800 rpm, 20 min, 24 °C (Figure S1). The PBMC were harvested at the interface layer, washed with PBS, and counted by cellmeter Auto T4 (Nexcelom Bioscience, USA). The percentage of low-density granulocytes (LDGs) were determined by PE Mouse Anti-Human CD14 (BD Biosciences, USA) and Percp-Cy5.5 Mouse Anti-Human CD15 (BD Biosciences, USA) staining (CD14^-/lo^CD15^+^).

Neutrophils were isolated from a red cell layer, followed by lysing buffer (BD Biosciences, USA) to remove red cells, washed with PBS, and counted. The purity of neutrophils was determined by CD16 staining (APC-conjugated anti-human CD16, BD Biosciences, USA) using flow cytometry (BD FASC arial II cytometer).

### Cell culture

The purity of neutrophils in the present study was all more than 96%. Freshly isolated neutrophils were resuspended in RPMI 1640 medium (Gibco, USA) combined with 10% fetal bovine serum (FBS, Gibco, USA) and seeded into the 24-well plates for 1^10^6^ per well with or without recombinant IFN-α (Novoprotein, China) for 4–16 h for the following experiments. As for the plasma stimulation experiment, we firstly pooled the plasma from 15 treatment-naïve pSS patients and 15 matched HCs together, then prepared RPMI 1640 medium (Gibco, USA) combined with 20% pSS or HC plasma, and then cultured with the freshly isolated neutrophils for 4 h. To further confirm the effect of rhIFN-α 2a on neutrophils, freshly isolated neutrophils were pretreated with IFN-α receptor inhibitor [IFN alpha-IFNAR-IN-1 hydrochloride, MedChemexpress (MCE), USA] for half an hour and then stimulated with rhIFN-α 2a for another 4 h.

### RNA sequencing (RNA-Seq) and data analysis

RNA sequencing analysis was performed in 7 pSS patients and 6 matched healthy controls. Total RNA of neutrophils was extracted using TRIzol reagent (Invitrogen, USA) and stored at −80 °C for subsequent RNA sequencing (Novogene, China). Raw data of fastq format were firstly processed through in-house per scripts, and clean data were obtained by removing reads containing adapter, reads containing poly-N, and low-quality reads. HTseq v0.6.0 was used to count the reads numbers mapped to each gene. Differential expression analysis was performed using DESeq2 R package (1.10.1). The resulting *P*-values were adjusted using the Benjamini and Hochberg’s approach for controlling the false discovery rate. Genes with an adjust *P*-value < 0.05 found by DESeq2 were defined as differentially expressed (DE).

Gene Ontology (GO) enrichment analysis of differentially expressed genes was implemented by the clusterProfiler R package, and GO terms with corrected *P*-value < 0.05 were considered significantly enriched by DE genes. KEGG (http://www.genome.jp/kegg/) is a database resource for understanding high-level functions and utilities of the biological system; clusterProfiler R package was also used to test the statistical enrichment of DE genes in KEGG pathways.

### Quantitative real-time PCR (RT-qPCR)

Total RNA was extracted using the RNA-Quick purification Kit (ES science, China), and complementary DNA (cDNA) synthesis was performed using Bestar qRCR RT Kit (DBI Biosciences, Germany) following the manufacturer’s instructions. Total RNA concentrations were measured by a Nanodrop2000c spectrophotometer (Nanodrop Technologies, USA). RT-PCR was performed using Bestar SybrGreen qPCR Mastermix (DBI Biosciences, Germany) and Roche LightCycler 480 II (Roche, Switzerland). Primer sequences are listed in Supplementary Table S1. The expression of gene expression was determined relative to β-Actin Forward primer 5′–3′ GGGACCTGACTGACTACCTC, Reverse primer TCATACTCCTGCTTGCTGAT by ΔΔCT method.

### Cell-free DNA (cf-DNA) detection

Concentration of plasma cf-DNA was measured by SYTOX Green Nuleic Acid Stain (Invitrogen, USA). One micromolar SYTOX Green combined with 200 μl plasma was added into 96-well black/clear polystyrene microplates (Corning, USA) and incubated at room temperature (RT) in dark for 5 min. The absorbance at 523 nm was measured using an Thermo Scientific Varioskan Flash (Thermo Fisher Scientific, USA).

### Myeloperoxidase (MPO) quantification

The MPO level in plasma or cell culture supernatant were measured using myeloperoxidase human ELISA Kit (Abcam ab119605, England) according to the manufacturer’s instructions. Briefly, 100 μl standards and 1:10 diluted samples were added to 96-well plates and incubated at 37 °C for 90 min. Then 100 μl 1 × Biotinylated anti-Human Myeloperoxidase antibody was added into each well and incubated at 37 °C for 60 min. After washing the plate 3 times with 0.01M PBS, 100 μL 1 × Avidin-Biotin-Peroxidase Complex working solution was added into each well and incubated at 37 °C for 30 min, the substrate (TMB color-developing agent) was then added, and absorbance at 450 nm was measured using a Thermo Scientific Multiskan FC (Thermo Fisher Scientific, USA).

### Detection of cytosolic ROS and mitochondrial ROS

Isolated neutrophils or LDGs were stained with ROS detection reagent, and the mean fluorescence intensity (MFI) was measured by flow cytometry. Cytosolic ROS production was measured by Fluorometric Intracellular Ros Kit (Sigma-Aldrich, USA), and mitochondrial ROS production was measured by MitoSOX (Invitrogen, USA) respectively.

### Immunofluorescence staining for NETosis markers

Labial glands from pSS patients and controls (patients with xerostomia and did not meet the diagnosis of primary Sjögren’s syndrome) were fixed in 4% tissue fixation solution (BD Biosciences, USA) and embedded in paraffin. DNA was stained with DAPI, myeloperoxidase (MPO), and citrullinated histone H3 (citH3) and was stained with anti-MPO primary antibody (Anti -Myeloperoxidase Mouse mAb, Servicebio, China), Rabbit Anti-human Histone H3 (citrulline R2 + R8 + R17, Abcam, USA), and goat anti-mouse/rabbit IgG secondary antibody.

### JC-1 staining for mitochondrial damage

Neutrophils were incubated with JC-1 working buffer (Solarbio, China) at 37 °C for 20 min and then washed with JC-1 buffer at 600 g, 5 min for two times. Flow cytometry was used to measure the mitochondrial stress.

### Statistical analysis

All the analysis in this study was conducted in SPSS (Version 22.0, IBM, Armonk, NY, USA), Microsoft Office Excel, and GraphPad prism (Version 8.0), and all the RNA-seq data was analyzed using R Statistical Package (Version 4.2.0, https://www.r-project.org). Data generated from this study would firstly be tested whether met the normal distribution and was described in mean ± S.D. or median (IQR) according to the normal distribution test. For the normally distributed data, Student’s *t* test was used, while the Mann-Whitney test was applied to the data meeting the non-normal distribution. Pearson correlation was used to find the correlation among patients’ clinical features and mRNAs, or the results of research related to NETosis. *P* < 0.05 was considered statistically significant.

## Results

### The increased NETting neutrophils in peripheral and salivary gland tissue of pSS patients

**Fig. 1:**
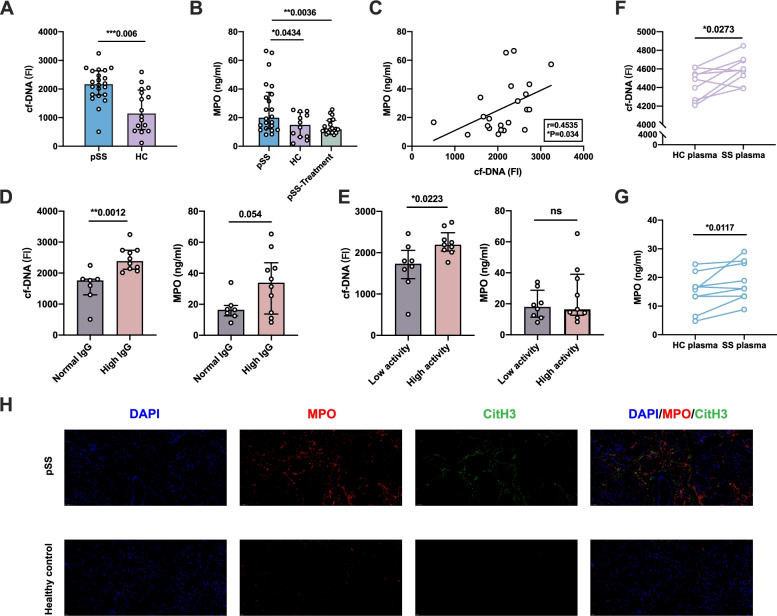
Enhanced NETosis markers in pSS patients. **A** Comparison of the fluorescence intensity (FI) of plasma cf-DNA between pSS patients (*n*=22) and matched healthy controls (*n*=16), each point represented the fluorescence intensity results of every subjects. **B** Comparison of the plasma MPO levels between pSS patients, matched healthy controls, and pSS patients after treatment, each point represented the fluorescence intensity results of every subject. **C** The correlation between plasma cf-DNA and MPO levels. **D** Comparison of plasma cf-DNA levels and MPO levels between patients with high IgG (*n*=10) or normal IgG (*n*=7) levels. **E** Comparison of Plasma cf-DNA levels and MPO levels between patients with high activity (*n*=9) or low activity (*n*=8), patients’ disease activity was assessed through ESSDAI (High activity, ESSDAI>5; low activity, ESSDAI≤5). **F,G** Comparison of plasma-stimulated neutrophils from pSS and HC to produce NETosis markers, **F** the fluorescence intensity of cf-DNA; **G** MPO production levels. **H** NETosis markers staining in pSS and HC labial glands (×40, blue for DAPI, red for MPO, green for CitH3). *P-value < 0.05, **P-value < 0.01, ***P-value <0.001)

We firstly explored whether pSS neutrophils exhibit increased NETosis by comparing the cf-DNA and MPO (two important NETosis markers) in plasma and labial gland between pSS patients and HCs. Both plasma cf-DNA and MPO levels were higher in pSS patients compared with HCs (cf-DNA: 2137.43±586.03 vs 1278.17±755.51, *P*=0.001; MPO: 26.98±18.12 vs 14.45±8.65, *P*=0.034, Fig. 1A,B). The positive correlation between FI of cf-DNA and MPO (*r*=0.4535, *P*=0.034, Fig. 1C) demonstrated the production of NETs in plasma and at least partly derived from neutrophils. Plasma cf-DNA levels were elevated in patients with high IgG (IgG > 18 g/L) (2456.81 ± 387.31 vs 1574.99 ± 549.32, *P*=0.0012, Fig. 1D), accompanied by the increasing MPO levels (33494.3 ± 19186.15 vs 17305.05 ± 8205.3, *P*=0.054). Similar results were also found in the patients with high disease activity (Fig. 1E), with higher cf-DNA levels (2237.66 ± 304.19 vs 1660.73 ± 583.53, *P*=0.0223) and the trend of higher MPO levels (25966.71 ± 18724.27 vs 19191.21 ± 9385.31, *P*=0.37). Plasma MPO levels were significantly decreased in stable pSS patients (patients who achieved stable disease status after treatment, *P*=0.0036, Fig. 1B). Moreover, neutrophils incubated with pSS patient-derived plasma could secrete higher cf-DNA and MPO than those incubated with HC-derived plasma (Fig. 1F,G). We further performed the immunofluorescence staining of DNA\MPO\citH3 and detected more infiltrating netting neutrophils (MPO\citH3) in labial gland of pSS patients compared with controls (Fig. 1H). Thus, the above results indicated the potential role of neutrophils and NETs in the pathogenesis of pSS.

### Neutrophils displayed a type I IFN signature in pSS patients

**Fig. 2:**
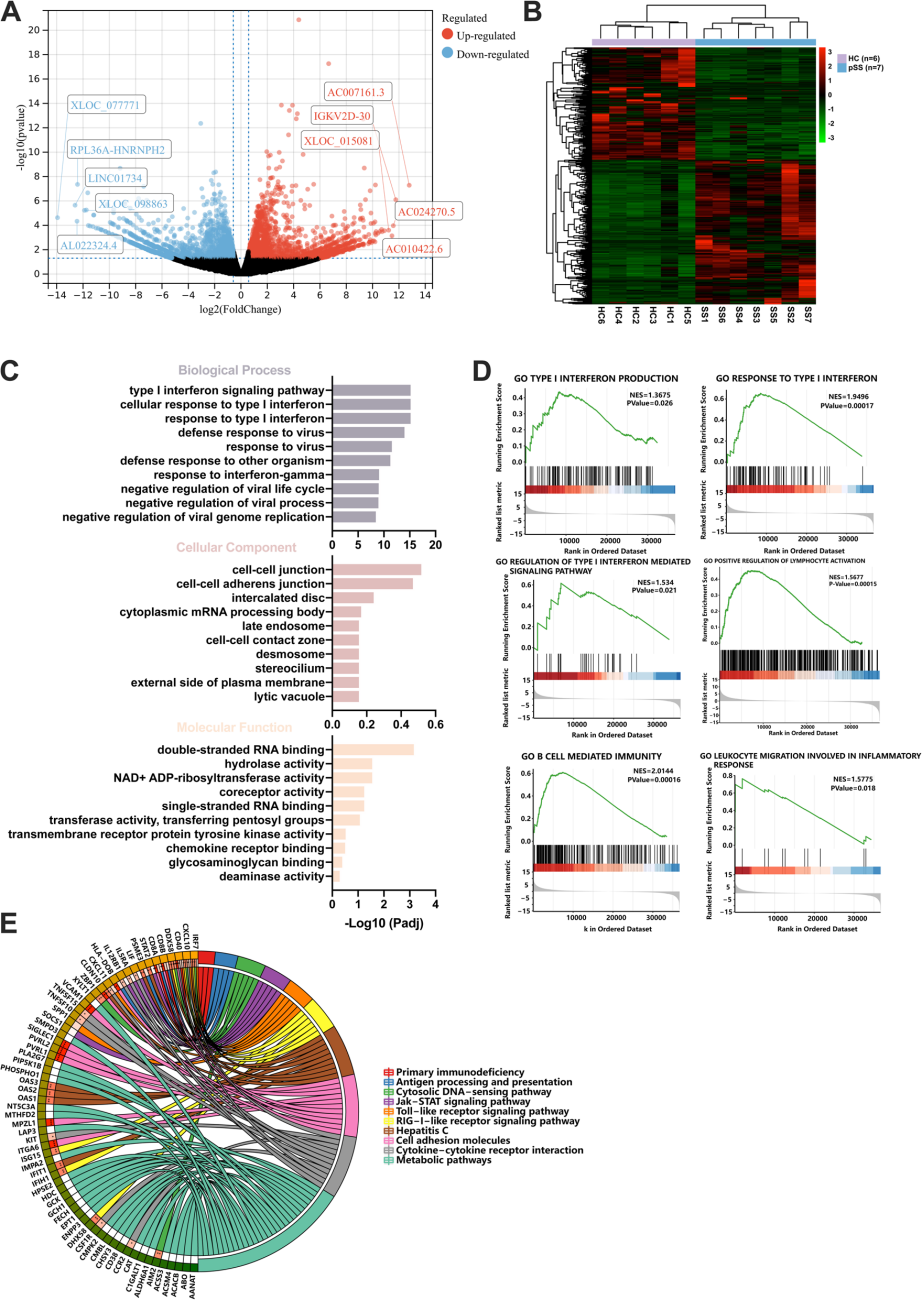
RNA-seq analysis for pSS neutrophils (pSS=7, HC=6). **A** The volcano plot for DE mRNAs of pSS neutrophils (239 upregulated in red and 181 downregulated mRNAs in blue, TOP 5 up- and downregulated genes were annotated). **B** The hierarchical clustering heatmap for DE mRNAs of the pSS neutrophils. **C** GO enrichment analysis for DE mRNAs of pSS neutrophils (implemented by the clusterProfiler R package). **D** GSEA for the potential function of DE mRNAs of pSS neutrophils. **E** KEGG analysis for the potential pathways of pSS neutrophils’ DE mRNAs. Different colors represented for the different signaling pathways. *P-value < 0.05, **P-value < 0.01, ***P-value <0.001)

To further explore the underlying molecular mechanisms in inducing NETosis of pSS neutrophils, we next performed RNA sequencing of 7 pSS patients and 6 sex-age matched healthy controls. We found a total of 239 upregulated mRNAs and 181 downregulated mRNAs and hierarchical clustering analysis of the DE mRNAs showed a distinct gene pattern of neutrophils in pSS patients compared with HCs (Fig. 2A,B). Functional enrichment analysis of DE mRNAs by Gene Ontology (GO) enrichment analysis revealed DE mRNAs of pSS neutrophils significantly enriched in type I interferon signaling pathway, response to type I interferon, and defense response to virus (Fig. 2C). Gene-set enrichment analysis (GSEA) showed that response to type I interferon (*P*=0.00017), type I interferon production (*P*=0.026), regulation of type I interferon mediated signaling pathway (*P*=0.021), positive regulation of lymphocyte activation (*P*=0.00015), B cell-mediated immunity (*P*=0.00016), and leukocyte migration involved in inflammatory response (*P*=0.018) were the significant function for pSS neutrophils (Fig. 2D). KEGG enrichment analysis indicated that DE mRNAs in pSS neutrophils also enriched in virus defense signaling pathways, cytosolic DNA-sensing pathway, antigen presentation, TLR signaling pathway, metabolic pathways, etc*.* (Fig. 2E).

**Fig. 3:**
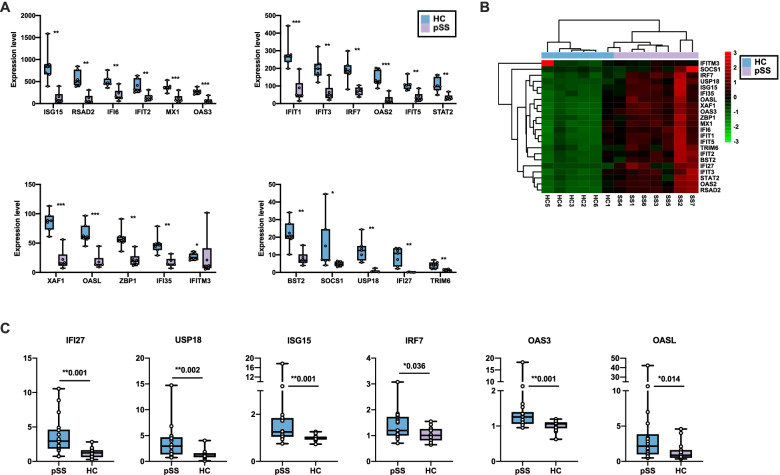
The overexpressed type I interferon signaling pathway-related genes in pSS neutrophils. **A** Comparison the expression level of type I-related genes between neutrophils isolated from pSS patients and healthy controls in RNA sequencing analysis (pSS=7, HC=6). **B** The hierarchical clustering heatmap for DE Type I-related genes in pSS and healthy neutrophils (pSS=7, HC=6). **C** RT-qPCR results for type I-related mRNAs in pSS and healthy neutrophils (pSS=18, HC=17). *P*-value > 0.05 indicated no statistically significant difference (ns), *P-value < 0.05, **P-value < 0.01, ***P-value <0.001

Furthermore, the expression levels of type I interferon signaling pathway-related genes including *ISG15, RSAD2, IFI6, IFIT2, MX1, OAS3, IFIT1, IFIT3, IFIT5, IRF7, OAS2, STAT2, XAF1, OASL, ZBP1, IFI35, IFITM3, BST2, SOCS1, USP18, IFI27*, and *TRIM6* were upregulated (Fig. 3A, Table S3), and the hierarchical clustering heatmap of these genes is shown in Fig. 3B. We then enrolled another 18 pSS patients and 16 age and sex-matched HCs and performed RT-PCR to confirm the upregulated type I interferon-related gene expression in neutrophils of pSS patients. We confirmed that mRNA expression of *IFI27* (*P*=0.001), *USP18* (*P*=0.002), *ISG15* (*P*=0.001), *IRF7* (*P*=0.036), *OAS3* (*P*=0.001), and *OASL* (*P*=0.014) were significantly elevated in pSS neutrophils compared with healthy neutrophils (Fig. 3C). Some type I IFN-related genes also had an increasing trend but with no statistical differences (Figure S2A-E). Collectively, our results demonstrated an overexpressed type I interferon signaling pathway-related genes in neutrophils from pSS patients.

### Excessive activation existed in pSS neutrophils

**Fig. 4:**
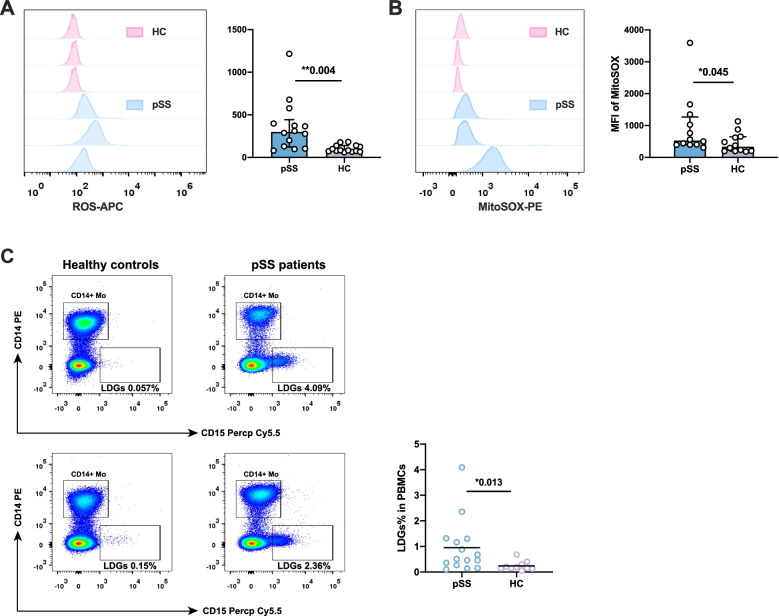
Measurements of neutrophil ROS production and LDGs percentage in pSS patients and healthy controls. **A** Representative flow cytometry results and comparison of ROS production in HC neutrophils (*n*=14) and pSS neutrophils (*n*=14). **B** Representative flowcytometry results and comparison of MiSox production in HC neutrophils (*n*=12) and pSS neutrophils (*n*=12). **C** Representative flowcytometry results and comparison of LDG percentage in PBMCs between pSS patients and healthy controls. *P-value < 0.05, **P-value < 0.01, ***P-value <0.001

Type I interferon was previously found to be a hallmark of pSS patients, which was found significantly elevated in the plasma and labial salivary gland of patients with pSS [16]. Combined with the type I IFN signature found in our study, we speculated that neutrophils might be activated by type I IFN in patients with pSS, while the activation of neutrophils and NET formation were accompanied by the ROS production. ROS production was reported to be an integral part of NETosis [17], which could also cause tissue damage. We further compared the intracellular ROS production in freshly isolated neutrophils from pSS and HCs. As shown in Fig. 4A, pSS neutrophils displayed a higher ROS production than healthy controls, with significantly elevated MFI of ROS measurement in pSS neutrophils (363.79±80.82 vs 103.67±11.15, *P*=0.004), which revealed a chronic oxidate stress and an activation state in pSS neutrophils, type I interferon might be at least partly the stimulating factor of pSS neutrophils.

Previous study also reported a distinct low-density granulocyte (LDG) as an immature neutrophil subtype with a stronger capacity to produce NETs and proinflammatory cytokines (IL-6, type I IFN, etc.) in some autoimmune diseases [18]. We also used CD14^lo/-^CD15^+^ as markers for LDG identification (Fig. 4C) [19] and found significantly elevated proportion of LDGs in PBMCs of pSS patients (0.95±0.27% vs 0.24±0.05%, *P*=0.014, Fig. 4C), with the significantly increasing ROS production than normal density neutrophils (Figure S3A-B). The stronger ability to secrete type I IFN and release NETs might further promote the activation of neutrophils in pSS patients.

### The effect of type I interferon induction on mitochondria damage in pSS neutrophils

**Fig. 5:**
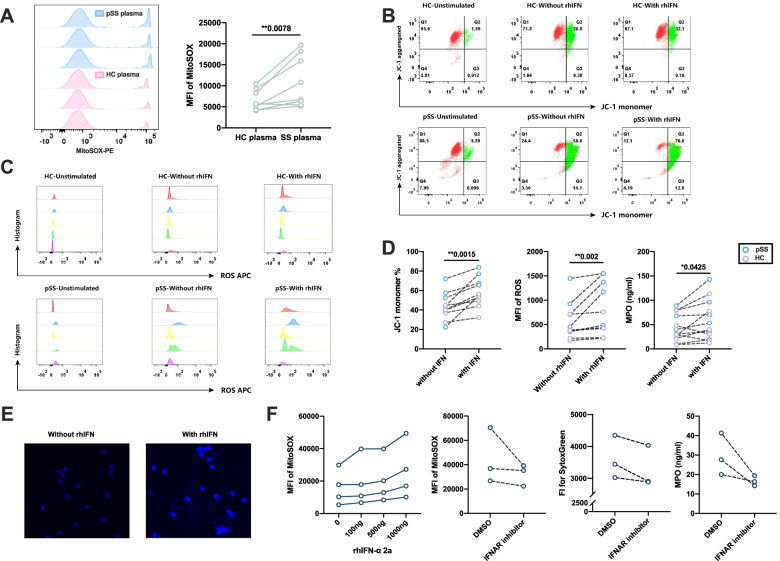
Results for rhIFN-α stimulation of neutrophils in pSS patients and healthy controls. **A** Results MitoSOX measurements of pSS and HC plasma stimulation of healthy neutrophils (*n*=9). **B** Representative flow cytometry results for the JC-1 staining in pSS patients and healthy controls with or without rhIFN stimulation (HC\pSS-Unstimulated represented for the freshly isolated neutrophils, pSS\HC-with or without rhIFN represented for the neutrophils stimulated with or without rhIFN). **C** Representative flow cytometry results of ROS production in pSS and healthy neutrophils with or without rhIFN stimulation (HC\pSS-Unstimulated represented for the freshly isolated neutrophils, pSS\HC-with or without rhIFN represented for the neutrophils stimulated with or without rhIFN, different color represented for the different subjects). **D** Comparison of JC-1 monomer percentage (mitochondrial damage, *n*=10), ROS production (*n*=10), and MPO concentration (*n*=12) between neutrophils stimulated with or without rhIFN-α. **E** Immunofluorescence staining of NETs-DNA (DAPI, blue) with or without rhIFN stimulation. **F** IFN-α receptor inhibitor reduced the mitochondrial damage (MitoSOX) and the production of NETs (SytocGreen and MPO levles). *P-value < 0.05, **P-value < 0.01, ***P-value <0.001)

Type I interferon was previously hypothesized to promote the mitochondrial damage and cause the mitochondrial ROS production in neutrophils [20], thereby inducing the NETosis of neutrophils. Thus, we subsequently performed the flow cytometry of MitoSOX in the freshly isolated neutrophils from pSS patients and healthy controls, and found an increasing MFI of MitoSOX in pSS neutrophils (951.5±934.86 vs 455.75±309.43, *P*=0.045, Fig. 4B). Through culturing neutrophils with pSS plasma and HC plasma to measure MitoSOX production, we found that pSS plasma promoted more mitochondrial ROS production in healthy neutrophils (*P*=0.0078, Fig. 5A). These results indicated the potential mitochondrial damage caused by type I IFN in pSS patients.

To elucidate the potential function of over-activated type I interferon signaling pathway in inducing mitochondrial damage and related ROS production of pSS neutrophils, we further stimulated isolated neutrophils from pSS patients and HCs using recombinant human interferon-α 2a (rhIFN-α 2a) in vitro. JC-1 was used to evaluate the high and low membrane potential of mitochondria through the JC-1 monomer percentage [21]. A high JC-1 monomer percentage indicated a low membrane potential and more mitochondrial damage. RhIFN-α could significantly cause MitoSOX production in healthy neutrophils in a dose-dependent manner, which could be inhibited by pretreatment with IFN receptor inhibitor (Fig. 5F). Comparing with the unstimulated neutrophils, neutrophils stimulated with rhIFN-α displayed a significant higher JC-1 monomer percentage (mitochondrial damage) (*P*=0.0015, Fig. 5B, D). And the effect of rhIFN-α stimulation was greater in pSS neutrophils (*P*=0.016, Figure S4A). RhIFN-α could also increase ROS production in neutrophils (*P*=0.002, Fig. 5C,D), and the effect on pSS neutrophils was also greater (*P*=0.016, Figure S4B). Consistently, MPO release and DAPI immunofluorescence was also significantly increased after rhIFN stimulation (59.09±41.71 vs 44.35±28.08, *P*=0.0425, Fig. 5D,E), and pSS neutrophils showed a tendency to produce NETs and to be more sensitive to type I interferon (Figure S4C). Pretreatment with IFN-α receptor inhibitor could significantly reduce the production of mitochondrial ROS, cf-DNA and MPO (Fig. 5F). Collectively, these results indicated that neutrophils could be activated by type I IFN through mitochondrial damage, and the activated neutrophils could produce ROS and release NETs to participate in disease pathogenesis, pSS neutrophils primed type I IFN seemed to be more sensitive to type I interferon.

## Discussion

In the present study, we found increased NETosis markers which were positively correlated with disease activity in pSS patients. We also detected infiltration of NETting neutrophils in pSS labial glands, suggesting the neutrophils and NETosis might be implicated in the tissue damage of pSS. Transcriptome analysis and RT-PCR confirmed type I IFN-induced gene signature in neutrophils from pSS patients. Further mechanism study in vitro revealed that type I IFN activation led to mitochondrial damage and related ROS production which finally result in the NET generation.

Aberrant NETosis or impaired NET clearance have been implicated in the pathogenesis of autoimmune diseases in previous studies [2, 22]. Both NETosis and NETs could play important roles in the initiation and amplification of autoimmune response. In AAV patients, autoantibodies against proteinase 3 (PR3) and MPO are important mechanisms in triggering disease response [23]. RA patient-derived NETs are found antigenic that could be specifically recognized by IgG autoantibodies from anti-citrullinated protein antibody (ACPA)-positive RA patients and activate macrophages [24]. The presence of NETs in SLE patients is an important source of SLE autoantigens and the resulting autoantibodies against NETs can inverse contribute to SLE pathogenesis [9, 25]. pSS as a well-known systemic autoimmune disease was manifested with B cell hyperactivity and autoantibodies (Anti-SSA, SSB, etc.). Our present study firstly demonstrated increased NETosis markers in plasma of pSS patients and positively associated with disease activity. We therefore hypothesized that autoantibodies in pSS might also have important mechanisms in triggering autoimmune responses in pSS patients. Besides, NET components like ROS or other granular proteins (NE, MPO, etc.) could also directly cause tissue damage. ROS production and HMGB1 could activate pDC through TLR7- or TLR9-signaling pathways and promote secretion of type I interferon [9]. LL-37 could promote the activation of NLRP3 and the release of proinflammatory cytokines (IL-1β, etc.) [26]. Whether the above mechanisms help promote the progression of inflammation response and direct tissue damage in pSS still needs further investigation.

High type I IFN was previously reported to be related to NETs formation [27]. Ersin Gul et al. [20] in their study confirmed that high type I interferon activity-related mitochondrial damage and NET formation might contribute to inflammatory manifestations in ataxia telangiectasia and artemis deficiency patients. Another study showed that blockade of IFN receptor (IFNAR) signaling using IFNAR-deficient mice or anti-IFNAR monoclonal antibodies (mAbs) abrogated NET formation with significantly fewer NET-forming neutrophils detected in the lung lesions of TB-susceptible mice, revealing an important role of type I IFN in inducing NET formation [28]. pSS was well-known as a type I interferon signature autoimmune disease with diverse upregulated interferon stimulated genes (ISGs) in both PBMCs and salivary gland biopsies [29–31]. In supplementary with previous studies, our present findings showed that pSS neutrophils displayed type I IFN signaling gene signatures. We also detected NETting neutrophils in pSS labial glands. Therefore, our present study highlighted the importance of type I IFN in promoting neutrophil-mediated peripheral and tissue inflammatory manifestations in pSS patients.

Our present study found significantly higher percentage and enhanced ROS production of LDGs in pSS patients. LDGs are a subset of proinflammatory neutrophils that were firstly identified in SLE patients [32]. Previous studies have reported enhanced capacity of NET production of LDGs than other neutrophil subsets in various diseases including pyogenic arthritis, pyoderma gangrenosum, and acne (PAPA) syndrome [33], idiopathic inflammatory myopathies (IIM) [34], and psoriasis [35]. They are also more immunostimulatory and interferogenic [36, 37]. Since we found NDGs from pSS patients displayed a type I interferon signature which contributed to NETosis, it would be an interesting topic to further discover whether pSS-derived LDGs are also more interferogenic and have an increased tendency to form NETs in vitro.

Classical NETosis are ROS-dependent and generation of ROS is considered to be critical for NETosis [17]. Mitochondria is one of the major sites of ROS generation, and mitochondrial ROS production could be sufficient to generate NETs in lupus [37]. Our present study found that ROS and MitoROS production increased in pSS neutrophils, indicating the potential NET formation in pSS neutrophils was ROS- and MitoROS-dependent. A type I interferon relate gene IFI27, which was found higher by RNA sequencing and RT-PCR validation, was reported to localize in mitochondria and might affect the mitochondrial electron transport chain gene expression [38]. Therefore, we speculated that neutrophils activated by type I interferon are accompanied by mitochondrial damage, ROS production, which leads to subsequent NETosis. To confirm the hypothesis, we further stimulated neutrophils from pSS patients with recombinant human IFN-α 2a and confirmed that type I IFN could induce mitochondrial damage and ROS production in neutrophils, especially pSS neutrophils. These results indicated the type I interferon-induced ROS and mitochondrial ROS production might be important mechanisms for pSS neutrophils.

Autoantibodies serve as one of the important class of stimuli in promoting the activation of neutrophils and the following formation of NETs [39–41] through MAPK pathway. Since the production of autobody and hyperglobulinemia was a hallmark of pSS, high levels of IgG in serum might also be another important source of the neutrophil activation in pSS patients, while the further experiments were still needed.

## Conclusions

**Fig. 6:**
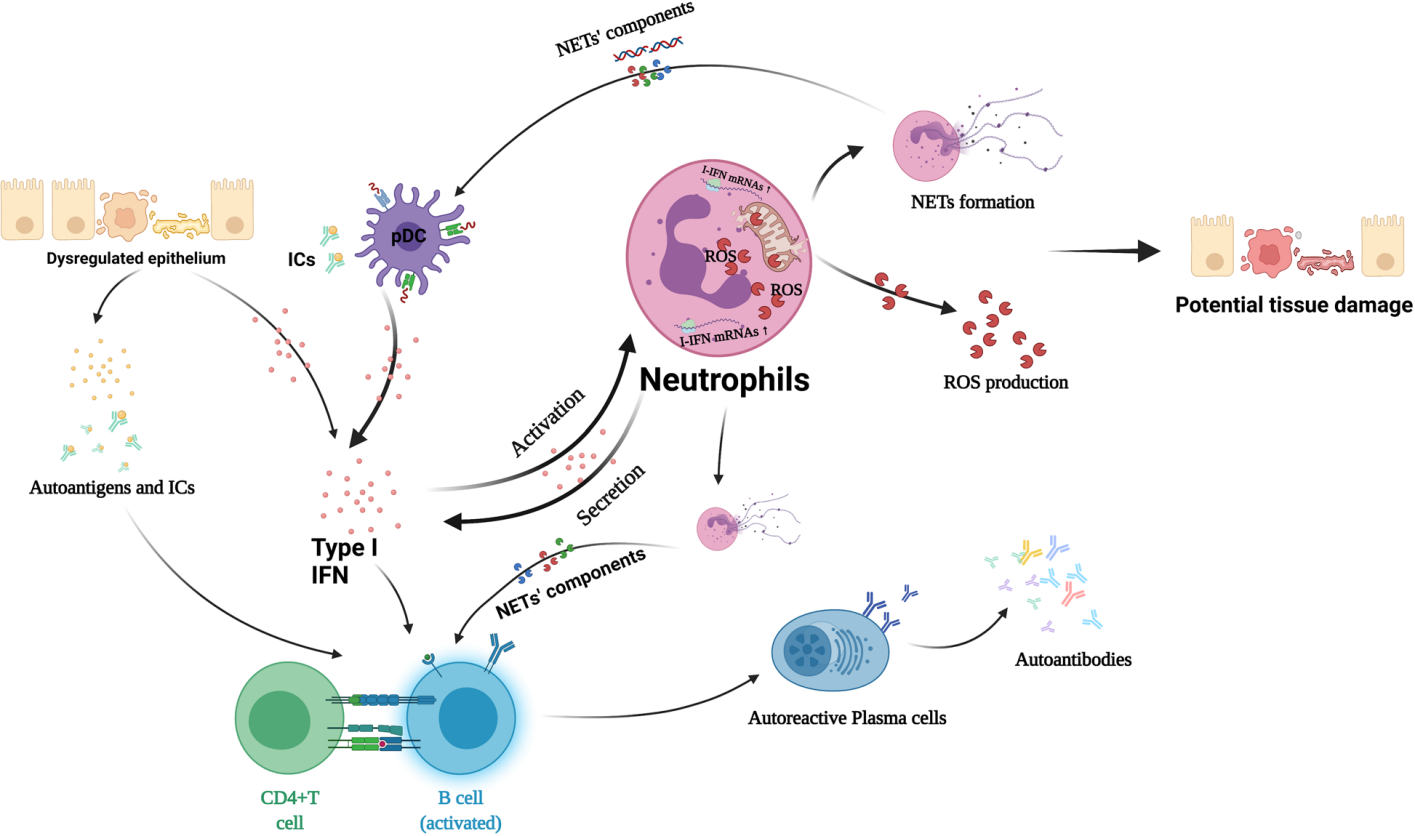
The potential role of neutrophils and neutrophil extracellular traps in the pathogenesis of primary Sjögren’s syndrome (Created with BioRender.com)

In summary, our present study demonstrated over-activated type I interferon signaling pathway induced ROS and mitochondrial ROS production and resulted in NETosis in pSS neutrophils. Importantly, we are the first to uncover the potential role of ROS-induced classical NETosis that contribute to inflammatory manifestations in pSS patients (Fig. 6). Thus, we inferred that anti-NET therapy might be a new target for clinical pSS therapy.

## Supplementary Information

**Additional file 1: Table S1**. The Primer used in RT-PCR analysis. **Table S2**. Top 10 up- and down-regulated mRNAs of pSS neutrophils in this study. **Table S3**. Statistics and comparison of the FPKM between pSS patients (*n*=7) and matched healthy controls (*n*=6). **Figure S1**. Process of obtaining the PBMCs and neutrophils from pSS patients and matched healthy controls (Created with BioRender.com). **Figure S2**. RT-qPCR results for type I related mRNAs in pSS and healthy neutrophils (pSS=18, HC=17). (*P*-Value > 0.05 indicated no statistically significant difference (ns), *P-Value < 0.05, **P-Value < 0.01, ***P-Value <0.001). **Figure S3**. The ROS production of LDGs. **Figure S4**. Comparison the stimulation results between pSS patients and HCs. (A) JC-1 monomer%; (B) MFI of ROS; (C) MPO levels. (*P-Value < 0.05, **P-Value < 0.01, ***P-Value <0.001).

## Data Availability

The raw sequence data reported in this study have been deposited in the Gene Expression Omnibus (GEO) under accession code (GSE194234) and are publicly accessible at http://www.ncbi.nlm.nih.gov/geo. Other data that support the findings of this study are available from the corresponding author upon reasonable request.

## Abbreviations

pSS: Primary Sjögren’s syndrome
IFN: Interferon
NETs: Neutrophil extracellular traps
rhIFN-α 2a: Recombinant human interferon-α 2a
ROS: Reactive oxygen species
mitoROS: Mitochondrial reactive oxygen species
cf-DNA: Cell-free DNA
MPO: Myeloperoxidase
LDG: Low-density granulocyte
ISG: Interferon stimulated gene
