# Supplementary material for: The potential roles of type I interferon activated neutrophils and neutrophil extracellular traps (NETs) in the pathogenesis of primary Sjögren’s syndrome
Source: Arthritis Res Ther. 2022 Jul 19;24:170. doi: 10.1186/s13075-022-02860-4 (PMC9295258; doi:10.1186/s13075-022-02860-4)
Supplement: Supplementary file 1 — Additional file 1: Table S1. The Primer used in RT-PCR analysis. Table S2. Top 10 up- and down-regulated mRNAs of pSS neutrophils in this study. Table S3. Statistics and comparison of the FPKM between pSS patients (n=7) and matched healthy controls (n=6). Figure S1. Process of obtaining the PBMCs and neutrophils from pSS patients and matched healthy controls (Created with BioRender.com). Figure S2. RT-qPCR results for type I related mRNAs in pSS and healthy neutrophils (pSS=18, HC=17). (P-Value > 0.05 indicated no statistically significant difference (ns), *P-Value < 0.05, **P-Value < 0.01, ***P-Value <0.001). Figure S3. The ROS production of LDGs. Figure S4. Comparison the stimulation results between pSS patients and HCs. (A) JC-1 monomer%; (B) MFI of ROS; (C) MPO levels. (*P-Value < 0.05, **P-Value < 0.01, ***P-Value <0.001). [file 13075_2022_2860_MOESM1_ESM.docx]

**Table S1. The Primer used in RT-PCR analysis.**

| **RNA** | **Forward primer (5’-3’)** | **Reverse primer (5’-3’)** |
| --- | --- | --- |
| ISG15 | GTTCATGAATCTGCGCCTGC | CAGCCTTTATTTCCGGCCCT |
| IRF7 | GAGATCCATACCGAGGCAGC | TGCCCTCTCAGGAGCCAA |
| IFI27 | TCCAAGCTTAAGACGGTGAGG | ATGGGCACAGCCACAACTC |
| USP18 | TTGGGCTCCTGAGGCAAATC | CAACCAGGCCATGAGGGTAG |
| IFIT1 | AGCAGGACCCACAAGAATGT | TAAGCTCAGCCTGATTGCGA |
| DDX58 | TGATACAAACTTGTACAGCCCAT | TCCCCTTTTGTCCTTGTGGG |
| DHX58 | GGCTCCTTCCCTCAGTTTCAG | TGGTAGGACCGAAGCTCCAT |
| STAT2 | GAAGCTGCACTTGGGAGTGA | GGATCCTGGGAAAAGGGCTG |
| OAS3 | GGAAGGACTCCCAGTTCAACA | GATGATAGGCCTGGGCTTCTG |
| OAS2 | GGAAAGTGCCGACAATGCAG | CACGGTGAGCCGAGTCTTTA |
| OASL | TTCGTGAAACATCGGCCAAC | GAGCATAGAGAGGGGGCAGA |

**Table S2. Top 10 up- and down-regulated mRNAs of pSS neutrophils in this study.**

| **Up-regulated mRNAs** | | | **Down-regulated mRNAs** | | | |
| --- | --- | --- | --- | --- | --- | --- |
| **Gene name** | **Log2 (Fold change)** | **P-value** | **Gene name** | **Log2 (Fold change)** | | **P-value** |
| L34079.1 | 10.90 | <0.001 | RPL36A-HNRNPH2 | | -12.42 | <0.001 |
| HPSE2 | 10.20 | <0.001 | AC117378.1 | | -11.75 | <0.001 |
| CHSY3 | 9.88 | <0.001 | MALRD1 | | -10.84 | <0.001 |
| VIT | 9.81 | <0.001 | FAM122A | | -9.94 | <0.001 |
| PTPRU | 9.37 | <0.001 | AC092647.5 | | -9.76 | <0.001 |
| TCERG1L | 9.34 | <0.001 | CDH6 | | -9.62 | <0.001 |
| PLA2G7 | 9.28 | <0.001 | IGLV10-54 | | -9.49 | <0.001 |
| MS4A4A | 9.11 | <0.001 | CADM2 | | -9.31 | <0.001 |
| RP1L1 | 9.07 | <0.001 | ACSM4 | | -9.24 | <0.001 |
| ADAMTS14 | 8.60 | <0.001 | AC005324.3 | | -8.92 | <0.001 |

**Table S3. Statistics and comparison of the FPKM between pSS patients (n=7) and matched healthy controls (n=6).**

| **I-IFN related mRNAs** | **pSS patients (n=7)** | **HC (n=6)** | **P-Value** |
| --- | --- | --- | --- |
| **ISG15** | 847.49±139.43 | 131.70±56.03 | **P=0.001 |
| **RSAD2** | 566.25±66.30 | 96.41±45.29 | ***P<0.001 |
| **IFI6** | 489.43±49.65 | 190.75±56.87 | **P=0.002 |
| **IFIT2** | 398.64±57.01 | 143.03±35.53 | **P=0.004 |
| **MX1** | 367.56±34.04 | 109.48±41.25 | ***P<0.001 |
| **OAS3** | 280.67±21.05 | 50.05±26.54 | ***P<0.001 |
| **IFIT1** | 279.90±28.96 | 73.19±26.47 | ***P<0.001 |
| **IFIT3** | 202.47±24.50 | 62.82±20.67 | **P=0.001 |
| **IRF7** | 189.13±24.55 | 69.84±9.78 | **P=0.001 |
| **OAS2** | 139.19±15.93 | 22.10±10.51 | ***P<0.001 |
| **IFIT5** | 107.70±11.48 | 37.38±10.61 | **P=0.001 |
| **STAT2** | 104.78±15.19 | 34.48±7.00 | **P=0.002 |
| **XAF1** | 86.70±6.29 | 21.70±7.16 | ***P<0.001 |
| **OASL** | 65.62±6.49 | 18.41±5.38 | ***P<0.001 |
| **ZBP1** | 58.35±6.32 | 21.53±4.80 | **P=0.001 |
| **IFI35** | 47.91±5.81 | 16.63±3.48 | **P=0.001 |
| **IFITM3** | 27.46±2.16 | 16.53±5.18 | *P=0.043 |
| **BST2** | 21.99±2.81 | 7.96±1.63 | **P=0.002 |
| **SOCS1** | 15.57±5.51 | 4.86±1.36 | *P=0.014 |
| **USP18** | 12.57±2.36 | 0.77±0.35 | **P=0.001 |
| **IFI27** | 9.08±1.84 | 0.12±0.05 | **P=0.001 |
| **TRIM6** | 4.22±0.70 | 1.09±0.28 | **P=0.003 |

**A P-Value > 0.05 indicated no statistically significant difference (ns), *P-Value < 0.05, **P-Value < 0.01, ***P-Value <0.001.**


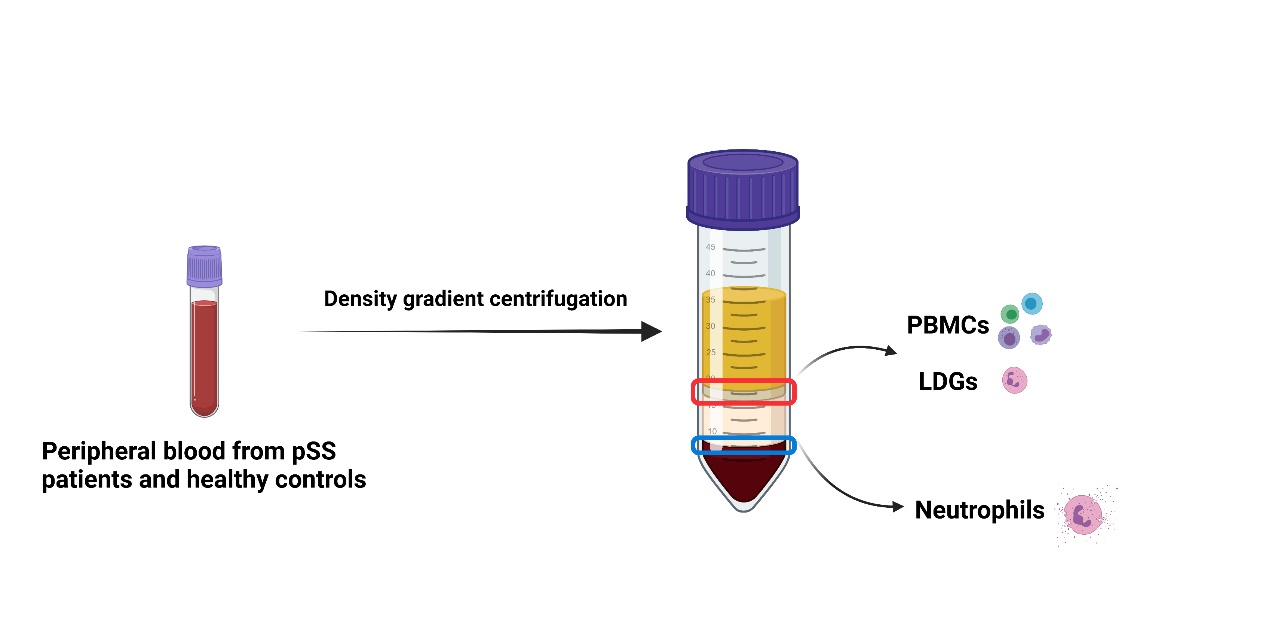


**Figure S1. Process of obtaining the PBMCs and neutrophils from pSS patients and matched healthy controls** **(Created with BioRender.com).**

**Figure S2. RT-qPCR results for type I related mRNAs in pSS and healthy neutrophils (pSS=18, HC=17). (P-Value > 0.05 indicated no statistically significant difference (ns), *P-Value < 0.05, **P-Value < 0.01, ***P-Value <0.001).**

**
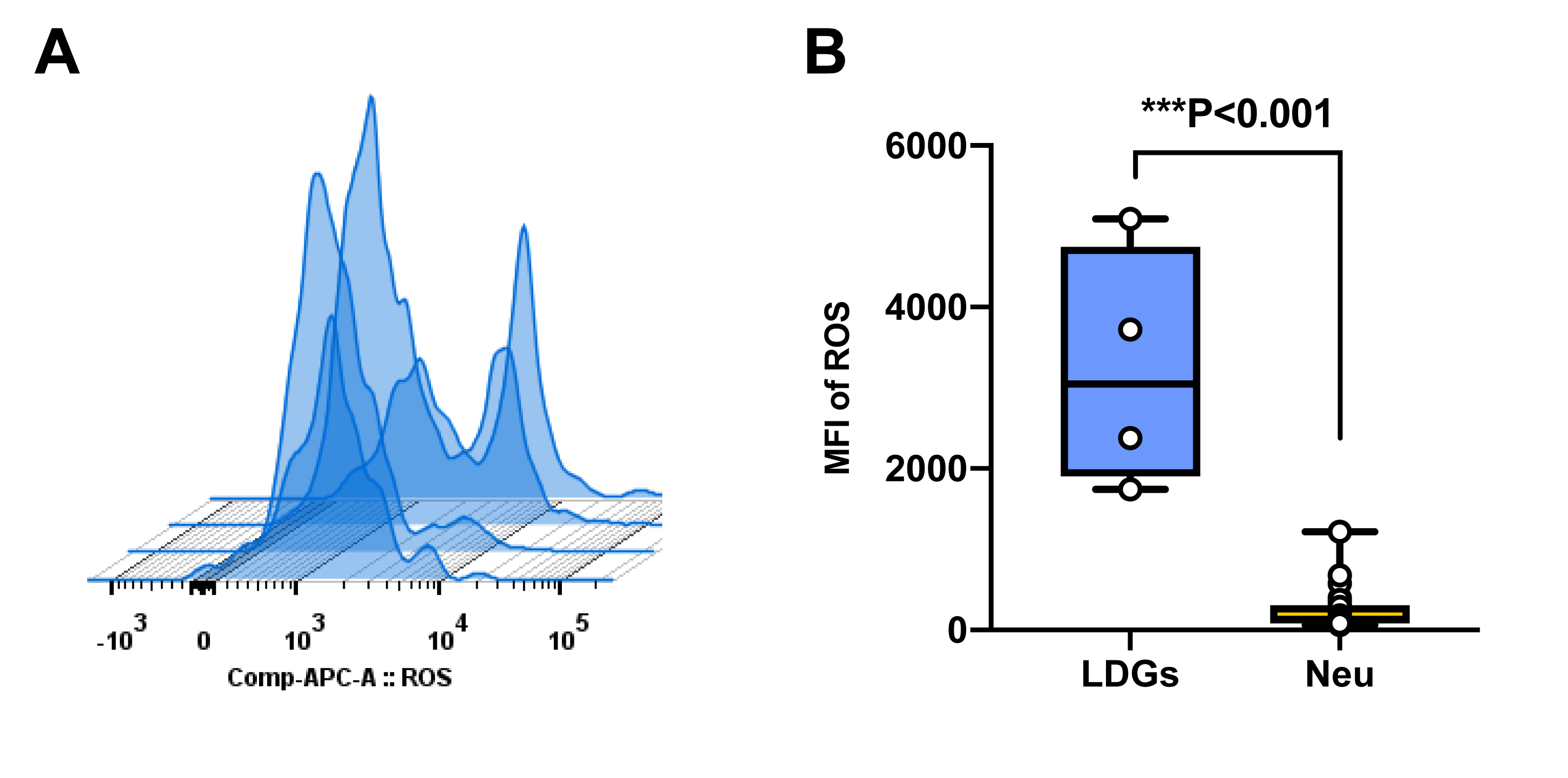
**

**Figure S3. The ROS production of LDGs.**

1. Flow cytometry analysis of ROS production for LDGs.
2. Comparison of the ROS production between LDGs and normal density granulocytes.

**Figure S4. Comparison the stimulation results between pSS patients and HCs. (A) JC-1 monomer%; (B) MFI of ROS; (C) MPO levels.** **(*P-Value < 0.05, **P-Value < 0.01, ***P-Value <0.001).**
